# Reproductive characteristics modify the association between global DNA methylation and breast cancer risk in a population-based sample of women

**DOI:** 10.1371/journal.pone.0210884

**Published:** 2019-02-14

**Authors:** Lindsay J. Collin, Lauren E. McCullough, Kathleen Conway, Alexandra J. White, Xinran Xu, Yoon Hee Cho, Sumitra Shantakumar, Susan L. Teitelbaum, Alfred I. Neugut, Regina M. Santella, Jia Chen, Marilie D. Gammon

**Affiliations:** 1 Department of Epidemiology, Emory University, Atlanta, GA, United States of America; 2 Department of Epidemiology, University of North Carolina, Chapel Hill, NC, United States of America; 3 Epidemiology Branch, National Institute of Environmental Health Science, Research Triangle Park, NC, United States of America; 4 Roche Product Development in Asia-Pacific, Shanghai, China; 5 Department of Biomedical and Pharmaceutical Sciences, University of Montana, Missoula, MT, United States of America; 6 Glaxo-Smith-Kline, Inc., Singapore; 7 Department of Preventive Medicine, Icahn School of Medicine at Mount Sinai, New York, NY, United States of America; 8 Department of Epidemiology, Columbia University, New York, NY,United States of America; 9 Department of Medicine, Columbia University, New York, NY, United States of America; 10 Department of Environmental Health, Columbia University, New York, NY, United States of America; 11 Department of Pediatrics, Icahn School of Medicine at Mount Sinai, New York, NY, United States of America; 12 Department of Oncological Science, Icahn School of Medicine at Mount Sinai, New York, NY, United States of America; University of South Alabama Mitchell Cancer Institute, UNITED STATES

## Abstract

DNA methylation has been implicated in breast cancer aetiology, but little is known about whether reproductive history and DNA methylation interact to influence carcinogenesis. This study examined modification of the association between global DNA methylation and breast cancer risk by reproductive characteristics. A population-based case-control study assessed reproductive history in an interviewer-administered questionnaire. Global DNA methylation was measured from white blood cell DNA using luminometric methylation assay (LUMA) and pyrosequencing assay (long interspersed elements-1 (LINE-1). We estimated adjusted odds ratios (ORs) and 95% confidence intervals (CIs) among 1 070 breast cancer cases and 1 110 population-based controls. Effect modification was assessed on additive and multiplicative scales. LUMA methylation was associated with elevated breast cancer risk across all strata (comparing the highest to the lowest quartile), but estimates were higher among women with age at menarche ≤12 years (OR = 2.87, 95%CI = 1.96–4.21) compared to >12 years (OR = 1.66, 95%CI = 1.20–2.29). We observed a 2-fold increase in the LUMA methylation-breast cancer association among women with age at first birth >23 years (OR = 2.62, 95%CI = 1.90–3.62) versus ≤23 years (OR = 1.32, 95% CI = 0.84–2.05). No modification was evident for parity or lactation. Age at menarche and age at first birth may be modifiers of the association between global DNA methylation and breast cancer risk.

## Introduction

Breast cancer remains the most commonly diagnosed invasive cancer among women in the United States [1]. Breast carcinogenesis is a multi-stage process involving both genetic and epigenetic changes [2], the latter defined as changes in gene expression, independent of modifications to the gene sequence [3]. Epigenetic aberrations have been implicated in breast carcinogenesis [4–6] and, unlike genetic alterations, these modifications may be altered across the lifespan by both exogenous and endogenous factors [7]. One commonly studied epigenetic modification is DNA methylation, characterized as the addition of a methyl group (-CH_3_) to the 5-carbon position of cytosine CpG dinucleotides [8]. DNA methylation is a regulatory mechanism for gene expression and may influence cancer development through activation or silencing of genes involved in tumorigenesis [8]. In addition to gene-specific DNA methylation, global DNA hypomethylation in regions that are normally methylated (i.e., tandem repeats or transposable elements) can lead to genomic instability and oncogene expression in breast tissue [8]. We previously reported that breast cancer risk was increased among women in the highest level of luminometric methylation assay (LUMA), but no association was observed when we considered long interspersed elements-1 (LINE-1) methylation in white blood cell (WBC) DNA [9].

Reproductive history, which comprises a group of well-established risk factors for breast cancer, contributes modestly to overall risk [10]; affecting the lifetime cumulative exposure of the breast epithelium to endogenous ovarian steroid hormones [11, 12]. Prior studies have shown that age at first birth and parity alter the methylation profile of CpGs related to ERα expression in non-malignant breast tissue [13]. Another previous investigation in 376 healthy women showed reproductive characteristics were inversely associated with markers of global methylation as measured by LUMA [14]. However, this study was limited to menarcheal age and did not explore the potential links to breast cancer incidence. While both reproductive characteristics and aberrant DNA methylation are known to be relevant to breast cancer, no previous research has investigated their potential interaction with respect to breast cancer risk. Cyclical DNA methylation/demethylation may be activated by steroid hormones [15], modulating the expression of steroid receptors. It is therefore biologically plausible that reproductive characteristics and DNA methylation have a joint effect on breast carcinogenesis.

In this current study, we sought to expand our previous work on the association between global DNA methylation and breast cancer risk by examining the potential modification of the association between global DNA methylation and breast cancer risk by age at menarche, age at first birth, parity, and lactation. We used two independent methods to assess global DNA methylation in WBC DNA. The first is a pyrosequencing assay, which measures methylation levels in repetitive, LINE-1; this method is commonly used as a surrogate marker of genome-wide DNA methylation [16, 17]. The second method, LUMA, measures the levels of 5-^m^C in the C^m^CGG motif which allows approximation of methylation levels at gene promoters rather than the total genome [9]. We also investigated whether reproductive characteristics were associated with global methylation in controls. We hypothesized that reproductive factors would modify the association between global DNA methylation and breast cancer risk.

## Materials and methods

Existing resources from the population-based case-control Long Island Breast Cancer Study Project (LIBCSP) were used to conduct our ancillary study. Details of participant recruitment, study design, and cohort characteristics have been described elsewhere [18]. All participating institutions obtained Institutional Review Board approval.

### Study population and study interview

Study participants in the LIBCSP were English-speaking female residents of Nassau and Suffolk counties of Long Island, New York [18]. Cases were defined as women with a newly diagnosed first primary breast cancer between August 1, 1996 and July 31, 1997, and were identified through daily or weekly contact with the local hospital pathology departments. Population-based controls were randomly selected using random digit dialling for women under age 65 and Health Care Finance Administration rosters for women ages 65 and older. Controls were frequency matched to the expected age distribution of case women by 5-year age groups.

**Fig 1:**
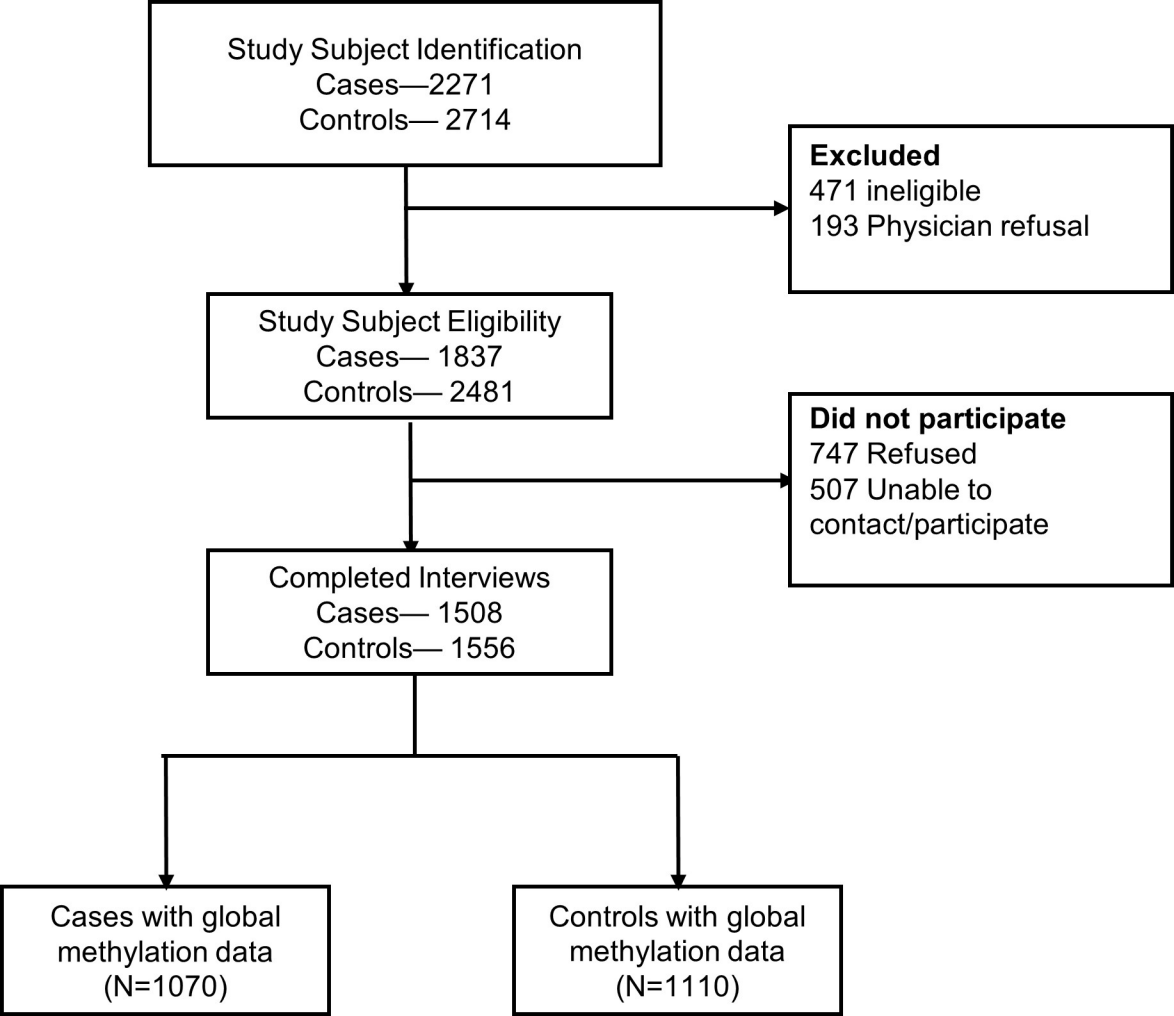
Selection and response of study population based on inclusion criteria of subjects in the population-based case control study of women age 20–98, in the Long Island Breast Cancer Study Project.

Interviews were completed for 82.1% (n = 1 508) of eligible cases and 62.8% (n = 1 556) of controls [18] (**Fig 1**). As abstracted from the medical/pathology records of the case women, 84.4% (n = 1 273) were diagnosed with invasive breast cancer, and 73.3% (of the 990 for whom this information was available) presented with estrogen receptor positive (ER+) breast cancer. Also, among all case-control participants, age at diagnosis (at identification for controls) ranged from 20 to 98 years, 67.1% were determined to be postmenopausal, and 92.8% were white (which is consistent with the underlying race distribution of the study counties at the time of data collection) [18] (**Table 1**). We previously reported that, among the LIBCSP population, increased breast cancer risk was associated with: early age at menarche, parity or nulliparity, late age at first birth, and little or no lactation [19]; and higher average lifetime alcohol intake [20]. Age-and menopausal-specific associations were also found for: use of oral contraceptives and hormone replacement therapy [21], adult weight gain [22]; increased body size and little or no physical activity [23, 24].

**Table 1:** Distribution of demographic and clinical characteristics by case-control status among the 1070 cases and 1110 controls with available global methylation data, Long Island Breast Cancer Study Project.

	Case (1070)	Control (1110)
	N (%)	N (%)
Age at diagnosis		
<50 years	294 (27.5)	371 (33.4)
≥50 years	776 (72.5)	739 (66.6)
Menopausal status		
Premenopausal	376 (35.1)	376 (33.9)
Postmenopausal	703 (65.7)	688 (66.1)
Missing	25	46
Cigarette Smoking		
Never	491 (45.9)	502 (45.3)
Current	205 (19.1)	209 (18.9)
Former	374 (35.0)	397 (35.8)
Missing	0	2
Age at menarche		
Less than 12 years	457 (43.1)	484 (43.9)
12 years or older	604 (56.9)	619 (56.1)
Missing	9	7
Parity		
Nulliparous	140 (13.1)	124 (11.2)
Parous	930 (86.9)	986 (88.8)
Age at first birth (among parous women only)		
Less than 23 years	314 (33.8)	355 (36.0)
23–27 years	310 (33.4)	345 (35.0)
Over 27 years	305 (32.8)	286 (29.0)
Missing	1	0
Lactation (among parous women only)		
Never	566 (60.9)	580 (58.8)
Any	364 (39.1)	406 (41.2)
ER Status		
Positive	422 (61.1)	
Negative	269 (38.9)	
Missing	379	

At the time of the case-control interview (which for cases occurred approximately two to three months after diagnosis), trained and licensed phlebotomists/nurses obtained a non-fasting blood sample from 73.1% of cases (N = 1 102) and 73.3% (N = 1 140) of control participants, which for most women occurred prior to adjuvant therapy. Study participants who agreed to donate a blood sample were generally younger than those who declined [18]. DNA was isolated from the donated blood samples using methods previously described [25].

Written informed consent was obtained from all participants prior to the study questionnaire, and prior to phlebotomy.

### Global methylation

Details of global methylation assessment within the LIBCSP study population have been described in detail [9]. Briefly, LUMA methylation levels were expressed as a percentage based on the following equation: methylation (%) = [1-(*HpaII* ∑G/∑T)/(*MspI* ∑G/∑T)]*100.[26] LINE-1 methylation levels were assessed using a previously validated pyrosequencing assay to assess 4 CpG sites in its promoter region [27, 28]. Methylation status at each of the four loci were analysed individual as a T/C single nucleotide polymorphism using QCpG software (Qiagen) and then averaged to provide an overall percentage 5^m^C status. As a quality control measure, to examine possible batch effects, samples were randomly selected for replication. Cases and controls were assayed simultaneously with the laboratory personnel blinded to both case-control status and quality control status. Of the 1 102 cases and 1 140 controls with available blood, 1 070 cases (97.1%) and 1 110 controls (97.4%) had valid global methylation data and were included in the study reported here.

### Reproductive characteristics

The reproductive characteristics of interest for our study were age at menarche, parity, age at first birth, and lactation. These variables were collected as part of the main 100-minute interviewer-administered questionnaire, which included a reproductive calendar to enhance recall [29]. For the purposes of this ancillary study, age at menarche was dichotomized at 12 years of age, and parity was categorized as nulliparous versus parous. Among parous women, age at first birth was dichotomized at 23 years of age, and women with any lactation were compared to women without lactation. These categorizations were based on previously published data in our study population [19], optimization of cell counts, and incorporation of biologic plausibility. We explored a three-level category for age at first birth (≤23, 23–27, ≥27 years of age), but observed similar effect estimates in the 23–27 and ≥27 categories. We therefore collapsed these two groups in our final analysis.

### Statistical methods

Using unconditional logistic regression we first estimated odds ratios (ORs) and 95% confidence intervals (CI) for the associations between reproductive characteristics and global methylation levels, among control women only (n = 1 110, 88.8% parous), given previous evidence that women with breast cancer (compared to women without breast cancer) are likely to have lower global methylation levels [30]. Then, using information from both cases and controls (2 180 women, 87.9% parous), we evaluated whether the association between global methylation and breast cancer risk was modified by reproductive history. Finally, as a sensitivity analysis, we also explored whether reproductive characteristics modified the association between global DNA methylation and ER+ breast cancer, given that sex-steroid hormones are a potential unifying mechanism for DNA methylation, reproductive characteristics and breast cancer risk [15].

We estimated the odds of breast cancer within quartiles of LUMA and LINE-1 global methylation markers and within strata of each of the four reproductive characteristics. For associations stratified by age at menarche and parity, models included all women (n = 2 180), but for age at first birth or lactation, models included parous women only (n = 1 916). Quartiles (Q) of LUMA and LINE-1 methylation were categorized based on distribution of the percent methylation among controls. Given that LUMA, a global measurement of promoter methylation, was positively associated with overall breast cancer risk in our study population (9), we used the lowest quartile as the referent category. In contrast, LINE-1 hypomethylation is hypothesized to be a marker of decreased genomic integrity, we therefore used the highest quartile of LINE-1 as the referent category.

Effect modification between global methylation and reproductive characteristics in association with breast cancer incidence was assessed on multiplicative and additive scales. Multiplicative interactions were evaluated using stratification and by comparing nested models with and without the interaction term using a Likelihood Ratio Test (*a priori* p-value <0.05). Additive interactions were evaluated using a common referent and the relative excess risk due to interaction (RERI) [31, 32] and corresponding 95% CIs calculated using the delta method [32].

For all models, potential confounders were first selected using directed acyclic graphs (DAGs). Potential covariates included: family history of breast cancer (no/yes), history of benign breast disease (no/yes), race (white/black/other), use of oral contraceptives (never/ever), body mass index (BMI, <25 kg/m^2^, 25–29.9 kg/m^2^, ≥30 kg/m^2^), recreational physical activity (RPA, high/moderate/inactive), alcohol history (never/ever), and smoking history (never/former/current). We further considered each possible confounder for inclusion in the final models based on a >10% change in estimate approach [33], but none met this criteria. Thus, final models presented here only include the frequency matching factor age (5-year age groups).

## Results

Among control women, we observed no associations between any reproductive characteristic and LUMA or LINE-1 methylation levels. Observed estimates were at or near the null, with all confidence intervals including the null (**S1 Table**).

### Age at menarche

As shown in **Table 2,** among women with age of menarche ≤12 years of age, breast cancer risk increased within each increasing quartile of LUMA methylation, compared to the lowest quartile (OR_Q2_ = 1.47, 95%CI = 0.98–2.20; OR_Q3_ = 2.23, 95%CI = 1.51–3.29; OR_Q4_ = 2.87, 95%CI = 1.96–4.21). A non-monotonic increase in breast cancer risk with increasing LUMA methylation was also observed among those with age at menarche >12 years of age, although the effect estimate was less pronounced (OR = 1.66, 95%CI = 1.20–2.29, comparing Q4 vs. Q1). This effect modification of the LUMA methylation-breast cancer association by age at menarche was evident on the multiplicative (p_interaction_ = 0.05), but not the additive scale [the RERI for additive interaction comparing those reporting an age at menarche ≤12 years and the highest level of global methylation assessed by LUMA (doubly exposed) to those with age at menarche >12 years and the lowest LUMA methylation levels (referent) was 0.19 (95%CI = -0.38–0.76)], [**S2 Table**].

**Table 2:** Age-adjusted odds ratios (ORs) and 95% confidence intervals (CIs) for the multiplicative interaction between the luminometric methylation assay (LUMA) and reproductive characteristics (age at menarche, age at first birth, parity, lactation) with breast cancer risk in a population-based sample of 2180 women with available global methylation data, Long Island Breast Cancer Study Project.

LUMA (Quartiles)	Cases/ Controls	OR	95% CI	Cases/ Controls	OR	95% CI
	**Age at Menarche**					
	>12 years			≤12 years		
Q1 (<0.43)	115/142	1.00	reference	66/131	1.00	reference
Q2 (0.43<0.56)	92/156	0.72	0.50–1.02	90/118	1.47	0.98–2.20
Q3 (0.56<0.66)	170/154	1.36	0.97–1.89	133/119	2.23	1.51–3.29
Q4 (≥0.66)	215/161	1.66	1.20–2.29	165/113	2.87	1.96–4.21
	*multiplicative p = 0*.*05*					
	**Parity**					
	Parous			Nulliparous		
Q1 (<0.43)	160/250	1.00	reference	23/26	1.00	reference
Q2 (0.43<0.56)	161/240	1.02	0.77–1.36	22/35	0.74	0.34–1.63
Q3 (0.56<0.66)	274/244	1.74	1.34–2.27	32/30	1.32	0.61–2.83
Q4 (≥0.66)	324/243	2.08	1.60–2.69	59/33	2.13	1.04–4.37
	*multiplicative p = 0*.*70*					
	**Age at First Birth**					
	≤23 years			>23 years		
Q1 (<0.43)	58/82	1.00	reference	102/168	1.00	reference
Q2 (0.43<0.56)	61/88	0.93	0.58–1.49	99/152	1.06	0.74–1.51
Q3 (0.56<0.66)	101/84	1.65	1.05–2.58	173/160	1.78	1.28–2.47
Q4 (≥0.66)	91/97	1.32	0.84–2.05	233/146	2.62	1.90–3.62
	*multiplicative p = 0*.*02*					
	**Lactation**					
	Any			Never		
Q1 (<0.43)	98/148	1.00	reference	62/102	1.00	reference
Q2 (0.43<0.56)	98/136	1.06	0.74–1.53	63/104	0.97	0.62–1.51
Q3 (0.56<0.66)	172/142	1.83	1.30–2.57	102/102	1.62	1.07–2.47
Q4 (≥0.66)	191/150	1.91	1.37–2.67	122/93	2.35	1.55–3.55
	*multiplicative p = 0*.*50*					

As shown in **Table 3,** the association between LINE-1 methylation and breast cancer risk (comparing Q1 vs. Q4) did not appear to differ between those with age at menarche ≤12 years of age (OR = 1.04, 95%CI = 0.72–1.49) and those who reported an age at menarche >12 years (OR = 1.08, 95%CI = 0.78–1.45).

**Table 3:** Age-adjusted odds ratios (ORs) and 95% confidence intervals (CIs) for the multiplicative interaction between the long-interspersed elements-1 (LINE-1) assay and reproductive characteristics (age at menarche, age at first birth, parity, lactation) with breast cancer risk in a population-based sample of 2180 women with available global methylation data, Long Island Breast Cancer Study Project.

LINE-1 (Quartiles)	Cases/ Controls	OR	95% CI	Cases/ Controls	OR	95% CI
	**Age at Menarche**					
	>12 years			≤12 years		
Q4 (≥80.4)	154/160	1.00	reference	123/116	1.00	reference
Q3 (78.7<80.4)	135/141	1.02	0.73–1.41	101/133	0.72	0.50–1.02
Q2 (77.0<78.7)	149/148	1.06	0.77–1.46	110/123	0.83	0.58–1.19
Q1 (<77.0)	162/163	1.06	0.78–1.45	122/110	1.04	0.72–1.49
	*multiplicative p = 0*.*43*					
	**Parity**					
	Parous			Nulliparous		
Q4 (≥80.4)	239/249	1.00	reference	40/27	1.00	reference
Q3 (78.7<80.4)	203/240	0.89	0.69–1.15	35/34	0.77	0.39–1.54
Q2 (77.0<78.7)	225/246	0.95	0.73–1.22	36/29	0.91	0.45–1.85
Q1 (<77.0)	257/243	1.12	0.87–1.44	29/32	0.62	0.30–1.26
	*multiplicative p = 0*.*41*					
	**Age at First Birth**					
	≤23 years			>23 years		
Q4 (≥80.4)	82/90	1.00	reference	156/159	1.00	reference
Q3 (78.7<80.4)	62/84	0.84	0.54–1.32	141/156	0.92	0.67–1.26
Q2 (77.0<78.7)	72/91	0.88	0.57–1.35	153/155	0.99	0.73–1.36
Q1 (<77.0)	96/88	1.25	0.82–1.90	161/155	1.06	0.67–1.26
	*multiplicative p = 0*.*75*					
	**Lactation**					
	Any			Never		
Q4 (≥80.4)	80/90	1.00	reference	159/159	1.00	reference
Q3 (78.7<80.4)	93/103	0.80	0.57–1.12	110/137	1.02	0.68–1.55
Q2 (77.0<78.7)	83/105	1.01	0.73–1.39	142/141	0.87	0.57–1.32
Q1 (<77.0)	107/105	1.11	0.80–1.52	150/138	1.15	0.77–1.73
	*multiplicative p = 0*.*53*					

### Parity

The association between LUMA methylation and breast cancer did not appear to differ substantially between strata of parity. When comparing the highest to the lowest quartile, we observed similar ORs between LUMA methylation and breast cancer risk among nulliparous (OR = 2.13, 95%CI = 1.04–4.37) and parous (OR = 2.08, 95%CI = 1.60–2.69) women (**Table 2**). Similarly, parity did not appear to modify the association between LINE-1 methylation and breast cancer risk (**Table 3**).

### Age at first birth

As shown in **Table 2**, among women with an age at first birth >23 years the risk of breast cancer increased with increasing quartiles of LUMA methylation (OR_Q2 vs Q1_ = 1.06, 95%CI = 0.74–1.51; OR_Q3 vs Q1_ = 1.78, 95%CI = 1.28–2.47; OR_Q4 vs Q1_ = 2.62, 95%CI = 1.90–3.62). By comparison, among women reporting early age at first birth (≤23 years) the association with breast cancer among women in the highest LUMA methylation quartile was less pronounced and included the null value (OR = 1.32, 95%CI = 0.84–2.05). This effect modification by age at first birth on the LUMA methylation-breast cancer association was evident on the multiplicative (p_interaction_ = 0.02) and additive scales [the observed RERI was 1.07 (95%CI = 0.43–1.71) comparing women with a first birth >23 years of age and the highest quartile of LUMA methylation (doubly exposed) to those with a first birth ≤23 years and the lowest quartile of LUMA methylation (referent).

Age at first birth did not appear to modify the association between LINE-1 methylation and breast cancer risk (**Table 3**). For women in the lowest quartile of LINE-1 methylation, there was a 25% increased risk of breast cancer within the strata of age at first birth ≤23 years, (OR = 1.25, 95%CI = 0.82–1.90) as compared with a 6% increased risk within the strata of age at first birth >23 (OR = 1.06, 95%CI = 0.67–1.26). We also considered a three-category variable (<23, 23–27, >27) for age at first birth, but found similar effect estimates among those with a first birth between 23–27 years of age and those >27 years of age (**S3 Table**).

### Lactation

The association between LUMA methylation and breast cancer risk was relatively homogenous across strata of lactation (**Table 2**). Comparing women in the highest quartile of LUMA methylation to the lowest, we observed around a 2-fold increase in the risk for breast cancer within the never lactating strata (OR = 2.35, 95%CI 1.55–3.55), as well as the ever lactating strata (OR = 1.91, 95%CI = 1.37–2.67) women. We additionally found no evidence for heterogeneity by lactation for the association between LINE-1 methylation and breast cancer (**Table 3**).

### Sensitivity analysis

In models restricted to ER+ breast cancer only, our results were similar to those shown, although the effect estimates were less precise (**S4 Table**).

## Discussion

In this population-based case-control study, we found that the association between global methylation, measured by LUMA in peripheral blood DNA, and breast cancer risk may depend on select reproductive characteristics, namely age at menarche and age at first birth (but not parity or lactation). Specifically, among women with age at menarche ≤12 years, there was nearly a 200% increase in breast cancer risk in association with high LUMA methylation levels; in contrast, the LUMA methylation-breast cancer association was only modestly increased by 66% among women with age at menarche >12 years (multiplicative interaction p = 0.05). Similarly, among women with a first birth >23 years, the association between high LUMA methylation levels and breast cancer risk was increased by greater than 150%; whereas among women with a first birth ≤23 years, the LUMA methylation-breast cancer association was increased by only 32% (multiplicative interaction p value = 0.02) No modification of the LINE-1 methylation-breast cancer association was observed by any of the four reproductive characteristics considered.

Numerous studies have been published on DNA methylation changes in breast tissue [34],^,^[35]. Less is known about WBC DNA methylation and breast cancer risk, although several studies have emerged. Given that blood biomarkers can be measured repeatedly over time in large populations, they represent a useful tool for uncovering aetiology and ultimately early detection [36]. To our knowledge, this is the first population-based study to examine modification of the association between global methylation and breast cancer risk by multiple reproductive characteristics using two independent global methylation assays measured in WBC. DNA hypomethylation (estimated via LINE-1) increases genomic instability leading to the activation of oncogenes, is likely to operate early in carcinogenesis, and is known to be affected by multiple factors (including age) [37]. In contrast, LUMA provides an overall quantitation of methylation levels at gene promoters [9] which may more directly reflect gene expression, cell proliferation, and other pathways important in cancer promotion or progression. The specificity of this marker may make it a better surrogate for understanding the role of well-defined exposures (*i*.*e*., reproductive characteristics) in cancer risk.

Contrary to our findings among control women, data from the EPIC-Italy sub-cohort showed that for each yearly increase in age at menarche, the odds of having genome-wide methylation (assessed by LUMA) below median level was increased by 32% (OR = 1.32, 95%CI = 1.14–1.53) [14]. The association between global methylation (assessed via LUMA) and breast cancer risk are mixed. While we previously estimated a 2.41-fold increased risk of breast cancer (OR_quintile 5 vs.1_ = 95%CI = 1.83–3.16) comparing quintile 5 vs. 1,[9] others report greater breast cancer risk among women in the lowest tertile of LUMA (OR = 2.86, 95%CI = 1.85–4.44, comparing tertile 1 vs. 3),[38] and still others found no association between measures by LUMA and breast cancer risk [39]. While the distribution of LUMA methylation levels in these studies may be an important consideration, the observed inconstancies highlight the importance of considering reproductive characteristics in combination with peripheral blood methylation and breast cancer [40]. Our data provide preliminary evidence that the presence of early menarche or late age at first birth, and elevated LUMA methylation may be particularly deleterious for breast cancer risk.

The divergence of the epigenome as a function of age due to stochastic changes in methylation (a phenomena termed “epigenetic drift”[41, 42]) may contribute to tumorigenic transformation [42] including chromosomal instability, mutations, genetic recombination, large deletions, or translocations [43]. This divergence may be influenced by different environmental and lifestyle factors throughout an individual’s life course [41]. Exposure of the genome to factors such as smoking and alcohol consumption have been shown to modify the biologic aging process [7]. Chronic stress has also been found to lead to epigenetic alterations and accelerated aging of tissue samples [44]. Furthermore, environmental and other factors, including hormonal changes during an individual’s lifespan, may contribute to the epigenetic alterations [41]. Our hypotheses that reproductive characteristics modify the association of DNA methylation and breast cancer risk relate to the conceptual framework that hormonal changes contribute to epigenetic drift. Our findings, particularly around age-related reproductive characteristics, support this hypothesis.

Our study has many strengths, namely the availability of global methylation assessments in a well-characterized population-based case-control study with a relatively large sample size. The study included detailed exposure assessment, particularly for reproductive history. Questionnaires were administered by trained interviewers using validated methods to mitigate recall error,[45] however, exposure was assessed retrospectively and may suffer from differential misclassification by case-control status. Nonetheless, it is unlikely that women knew their methylation status and thus any differential recall of reproductive characteristics would not likely bias the interaction parameters. Because global methylation was assessed in cases shortly after diagnosis, there is concern for reverse causality. However, in a nested case-control study, investigators of the Sister Study Cohort showed that aberrant DNA methylation (assessed in blood) was present in women some 5 years prior to diagnosis and was predictive of breast cancer risk [46]. Another consideration of the current study is the racial and ethnic homogeneity of our study population. This limits the generalizability of our findings to primarily white women, who are at the highest risk of developing breast cancer in the United States [47]. Further research in prospective studies with diverse study populations is needed to confirm our findings.

## Conclusions

Using resources from a large population-based case-control study, we observed that high methylation levels, assessed using the LUMA platform, were differentially associated with breast cancer risk among women with an earlier age at menarche and a later age at first birth. This study provides etiologic insight into how age-related reproductive factors may influence breast cancer risk through its interaction with the DNA methylome. Our findings may also help in the identification of an early biomarker among women who are at an increased risk of breast cancer based on their reproductive history.

## Supporting information

S1 Table Evaluation of the association between reproductive characteristics and DNA methylation among controls. (DOCX)

S2 Table Evaluation of the modification of the effect of DNA methylation on breast cancer risk by reproductive characteristics on the additive scale. (DOCX)

S3 Table Use of three category age at first birth to evaluate effect modification of the association between DNA methylation and breast cancer risk. (DOCX)

S4 Table Sensitivity analysis of the modification of the effect of DNA methylation on breast cancer risk among ER+ breast cancer cases. (DOCX)
