# Supplementary material for: Reproductive characteristics modify the association between global DNA methylation and breast cancer risk in a population-based sample of women
Source: PLoS One. 2019 Feb 14;14(2):e0210884. doi: 10.1371/journal.pone.0210884 (PMC6375664; doi:10.1371/journal.pone.0210884)
Supplement: S1 Table — (DOCX) [file pone.0210884.s001.docx]

**S1 Table:** Age-adjusted association between reproductive characteristics (age at menarche, age at first birth, parity, and lactation) and LUMA or LINE-1 methylation among 1110 population-based control women in the Long Island Breast Cancer Study Project.

|  | **Age at Menarche** | | | | | | | | |
| --- | --- | --- | --- | --- | --- | --- | --- | --- | --- |
| **LUMA (Quartiles)** | >12 years | ≤12 years | OR | 95% CI | **LINE-1 (Quartiles)** | >12 years | ≤12 years | OR | 95% CI |
| Q1 (<0.43) | 142 | 131 | 1.00 | Reference | Q4 (≥80.4) | 160 | 110 | 1.00 | Reference |
| Q2 (0.43<0.56) | 156 | 118 | 0.82 | 0.54-1.07 | Q3 (78.7<80.4) | 148 | 123 | 1.30 | 0.93-1.82 |
| Q3 (0.56<0.66) | 154 | 119 | 0.84 | 0.60-1.17 | Q2 (77.0<78.7) | 141 | 133 | 1.15 | 0.82-1.61 |
| Q4 (≥0.66) | 161 | 113 | 0.76 | 0.54-1.07 | Q1 (<77.0) | 160 | 116 | 0.93 | 0.66-1.30 |
|  | **Parity** | | | | | | | | |
|  | Parous | Nulliparous | OR | 95% CI |  | Parous | Nulliparous | OR | 95% CI |
| Q1 (<0.43) | 250 | 26 | 1.00 | Reference | Q4 (≥80.4) | 249 | 27 | 1.00 | Reference |
| Q2 (0.43<0.56) | 240 | 35 | 1.42 | 0.83-2.45 | Q3 (78.7<80.4) | 240 | 34 | 1.26 | 0.73-2.17 |
| Q3 (0.56<0.66) | 244 | 30 | 1.18 | 0.68-2.07 | Q2 (77.0<78.7) | 246 | 29 | 1.08 | 0.62-1.89 |
| Q4 (≥0.66) | 243 | 33 | 1.25 | 0.72-2.16 | Q1 (<77.0) | 243 | 32 | 1.12 | 0.65-1.89 |
|  | **Age at First Birth** | | | | | | | | |
|  | ≤23 years | >23 years | OR | 95% CI |  | ≤23 years | >23 years | OR | 95% CI |
| Q1 (<0.43) | 82 | 168 | 1.00 | Reference | Q4 (≥80.4) | 90 | 159 | 1.00 | Reference |
| Q2 (0.43<0.56) | 88 | 152 | 0.84 | 0.58-1.22 | Q3 (78.7<80.4) | 84 | 156 | 1.05 | 0.83-1.52 |
| Q3 (0.56<0.66) | 84 | 160 | 0.93 | 0.64-1.35 | Q2 (77.0<78.7) | 91 | 155 | 0.96 | 0.67-1.39 |
| Q4 (≥0.66) | 97 | 146 | 0.73 | 0.51-1.06 | Q1 (<77.0) | 88 | 155 | 0.99 | 0.69-1.44 |
|  | **Lactation** | | | | | | | | |
|  | Any | Never | OR | 95% CI |  | Any | Never | OR | 95% CI |
| Q1 (<0.43) | 148 | 102 | 1.00 | Reference | Q4 (≥80.4) | 98/148 | 62/102 | 1.00 | Reference |
| Q2 (0.43<0.56) | 136 | 104 | 0.89 | 0.62-1.28 | Q3 (78.7<80.4) | 98/136 | 63/104 | 0.76 | 0.52-1.09 |
| Q3 (0.56<0.66) | 142 | 102 | 0.95 | 0.66-1.36 | Q2 (77.0<78.7) | 172/142 | 102/102 | 0.75 | 0.53-1.09 |
| Q4 (≥0.66) | 150 | 93 | 1.13 | 0.79-1.63 | Q1 (<77.0) | 191/150 | 122/93 | 0.76 | 0.54-1.09 |
