# Supplementary material for: Reproductive characteristics modify the association between global DNA methylation and breast cancer risk in a population-based sample of women
Source: PLoS One. 2019 Feb 14;14(2):e0210884. doi: 10.1371/journal.pone.0210884 (PMC6375664; doi:10.1371/journal.pone.0210884)
Supplement: S2 Table — (DOCX) [file pone.0210884.s002.docx]

**S2 Table**. Age-adjusted odds ratios (ORs) and 95% confidence intervals (CIs) for the additive interaction between the luminometric methylation assay (LUMA), long-interspersed elements-1 (LINE-1) and age at first birth with breast cancer risk in a population-based sample of 2180 women with available global methylation data, Long Island Breast Cancer Study Project.

|  | **Age at First Birth Categories** | | | | | | | | |
| --- | --- | --- | --- | --- | --- | --- | --- | --- | --- |
| *LINE-1* | ≤23 | | | 23–27 | | | ≥27 | | |
| Q1 | 96/88 | 1.25 | 0.82–1.90 | 79/94 | 0.89 | 0.58-1.35 | 82/61 | 1.32 | 0.83-2.12 |
| Q2 | 72/91 | 0.88 | 0.57–1.35 | 69/83 | 0.84 | 0.54-1.31 | 84/72 | 1.13 | 0.72-1.79 |
| Q3 | 62/84 | 0.84 | 0.54–1.32 | 74/74 | 1.07 | 0.69-1.66 | 67/82 | 0.79 | 0.49-1.25 |
| Q4 | 82/90 | 1.00 | reference | 85/90 | 1.00 | reference | 71/69 | 1.00 | reference |
| *LUMA* | ≤23 | | | 23–27 | | | ≥27 | | |
| Q1 | 58/82 | 1 | reference | 49/89 | 1.00 | reference | 53/79 | 1.00 | reference |
| Q2 | 61/88 | 0.93 | 0.58–1.49 | 56/84 | 1.19 | 0.73-1.94 | 43/68 | 0.93 | 0.55-1.56 |
| Q3 | 101/84 | 1.65 | 1.05–2.58 | 85/91 | 1.70 | 1.07-2.69 | 88/69 | 1.90 | 1.19-3.05 |
| Q4 | 91/97 | 1.32 | 0.84–2.05 | 116/80 | 2.54 | 1.61-3.99 | 117/66 | 2.71 | 1.70-4.30 |
