# Supplementary material for: Reproductive characteristics modify the association between global DNA methylation and breast cancer risk in a population-based sample of women
Source: PLoS One. 2019 Feb 14;14(2):e0210884. doi: 10.1371/journal.pone.0210884 (PMC6375664; doi:10.1371/journal.pone.0210884)
Supplement: S4 Table — (DOCX) [file pone.0210884.s004.docx]

| **LUMA (Quartiles)** | Cases/ Controls | OR | 95% CI | Cases/ Controls | OR | 95% CI |
| --- | --- | --- | --- | --- | --- | --- |
|  | **Age at Menarche** | | | | | |
|  | >12 years | | | ≤12 years | | |
| Q1 (<0.43) | 48/142 | 1.00 | reference | 27/131 | 1.00 | reference |
| Q2 (0.43<0.56) | 41/156 | 0.77 | 0.48-1.24 | 46/118 | 1.85 | 1.08-3.17 |
| Q3 (0.56<0.66) | 74/154 | 1.39 | 0.90-2.15 | 47/119 | 1.90 | 1.11-3.25 |
| Q4 (≥0.66) | 78/161 | 1.43 | 0.93-2.19 | 55/113 | 2.35 | 1.39-3.98 |
|  | **Parity** | | | | | |
|  | Parous | | | Nulliparous | | |
| Q1 (<0.43) | 67/250 | 1.00 | reference | 9/26 | 1.00 | reference |
| Q2 (0.43<0.56) | 75/240 | 1.13 | 0.78-1.65 | 12/35 | 1.08 | 0.39-2.98 |
| Q3 (0.56<0.66) | 111/244 | 1.64 | 1.16-2.34 | 11/30 | 1.21 | 0.42-3.43 |
| Q4 (≥0.66) | 110/243 | 1.66 | 1.17-2.37 | 24/33 | 2.29 | 0.90-5.85 |
|  | **Age at First Birth** | | | | | |
|  | ≤23 years | | | >23 years | | |
| Q1 (<0.43) | 27/82 | 1.00 | reference | 40/168 | 1.00 | reference |
| Q2 (0.43<0.56) | 29/88 | 0.96 | 0.52-1.76 | 45/152 | 1.22 | 0.75-1.98 |
| Q3 (0.56<0.66) | 45/84 | 1.58 | 0.89-2.79 | 66/160 | 1.68 | 1.07-2.64 |
| Q4 (≥0.66) | 26/97 | 0.8 | 0.43-1.48 | 84/146 | 2.39 | 1.54-3.71 |
|  | **Lactation** | | | | | |
|  | Any | | | Never | | |
| Q1 (<0.43) | 22/102 | 1.00 | reference | 45/148 | 1.00 | reference |
| Q2 (0.43<0.56) | 28/104 | 1.24 | 0.66-2.31 | 47/136 | 1.09 | 0.68-1.74 |
| Q3 (0.56<0.66) | 46/102 | 1.98 | 1.11-3.55 | 65/142 | 1.48 | 0.95-2.31 |
| Q4 (≥0.66) | 43/93 | 2.09 | 1.16-3.77 | 67/150 | 1.45 | 0.93-2.27 |

**S4 Table.** Age-adjusted odds ratios (ORs) and 95% confidence intervals (CIs) for the association between the luminometric methylation assay (LUMA) and reproductive characteristics (age at menarche, age at first birth, parity, lactation) with estrogen receptor positive breast cancer risk in a population-based sample of 1532 women with available global methylation data, Long Island Breast Cancer Study Project.
